# Insights into Oral and Written Competencies in Neurodevelopmental Disorders

**DOI:** 10.3390/brainsci14020163

**Published:** 2024-02-06

**Authors:** Sergio Melogno, Maria Antonietta Pinto, Mila Vulchanova

**Affiliations:** 1 Department of Psychology of Development and Socialization Processes, “Sapienza” University of Rome, 00185 Rome, Italy; mariantonietta.pinto@uniroma1.it; 2 Faculty of Psychology, “Niccolò Cusano” University of Rome, 00166 Rome, Italy; 3 Language Acquisition and Language Processing Lab, Norwegian University of Science and Technology, 7491 Trondheim, Norway; mila.vulchanova@ntnu.no

## 1. Introduction

The study of language abilities offers privileged insights to access the multifaceted world of neurodevelopmental disorders (NDD, henceforth), showing how particular aspects of language may be handled differently as a function of typical neuropsychological features of specific disorders. 

The connection between two complex areas—neurodevelopmental disorders, on the one hand, and language, on the other, in both its oral and written forms—has recently generated several strands of research which further complicate the field. Within the limitations of this editorial, we will indicate just some of these strands which all challenge, more or less intentionally, the diagnostic criteria of well-established nosographic categories and, as a consequence, also the boundaries between those categories. The position that most explicitly calls into question the diagnostic criteria of well-known NDDs goes under the suggestive name of “trans-diagnostic revolution” [1]. This position is based on the observation that some symptoms have a transversal character across broad NDD categories and, at the same time, there might be remarkable differences at cognitive and behavioral levels under the same label [2,3]. dIn the words of the authors of an article highly representative of this current of thought [1], “A transdiagnostic approach to neurodevelopment is more than alternative sampling frames and sophisticated analysis methods, valuable as they are. It reflects a deeper reconceptualisation of the nature of NDD” [ibidem, p. 407, our italics]. Instead of searching for a common causal mechanism accounting for all observed profiles within a diagnostic category [4,5,6], a transdiagnostic perspective looks at transversal dimensions and/or possible clusters of dimensions. The abovementioned authors, for instance, noticed that executive functioning runs across such conditions as ADHD, Conduct Disorder, Oppositional Defiant Disorder, autism, and children without diagnosed conditions [7,8,9,10,11,12,13].

Other researchers, although accepting the canonical NDD classifications, tried to dig into a given NDD to see whether there might be some subtypes so far underinvestigated and comparable to other phenotypes, which is also a way to rethink the boundaries within and across disorder categories. An emblematic example of this approach is the study by Whitehouse, Barry, and Bishop [14], where children with specific language impairment (SLI, henceforth) were compared to children with autism and poor structural language abilities (Apoor, henceforth) and children with autism and appropriate language abilities (Aapp, henceforth) on nonword repetition performance, which is considered a typical weakness in SLI [15,16]. The results showed that, beyond some similarities between the low scores in nonword repetition in the SLI and Apoor groups, there were clear qualitative differences in the respective profiles of errors. More importantly, multiple weaknesses in language abilities in the Apoor group were associated with more severe autistic symptomatology than in the Aapp group. Taken together, these results suggest the implausibility of an SLI subtype within autism.

Lastly, other authors critically analyze the consistency of the positive outcomes of interventions on language abilities in populations with DDNs. A case in point is the recent meta-analysis by Donolato, Toffalini, Rodge, Anders, Nordhal-Hansen, Lerväg, Norbury, Melby-Lerväg [17], based on 38 studies conducted through interventions on a wide range of language abilities in children from 2 to 18 years with Developmental Language Disorder, autism, intellectual disability, Down Syndrome, Fragile X Syndrome, and Williams syndrome. The results showed encouraging effect sizes at post-test and follow-up, which, once again, are likely to significantly reshape our views of the language competencies of several DDN categories and, more importantly, of their potentialities.

## 2. In this Special Issue

The articles included in this Special Issue this Special Issue also address language issues related to categories of DDNs from novel perspectives, either by exploring new connections between a given DDN category and some cognitive or linguistic dimension, or by creating more nuanced degrees of deficient competencies within a given DDN category, in which case further unexplored dimensions enter into play, or else by capturing the potentialities that might emerge with facilitated tasks. This is the case for the first four articles, two of which focus on dyslexia, which pertains to learning disabilities, while the two others focus on William syndrome (WS, henceforth), which pertains to genetic disorders. A complimentary although more indirect contribution to our topic comes from two studies on metalinguistic abilities in typically developing children. Qualitative and contextual aspects of developmental trajectories are highlighted in these children, which helps to create a less discontinuous, all-or-none picture of language competencies compared to atypically developing populations. 

One of the studies on dyslexia (Contribution 1), from Aix–Marseille and Lyon Universities (France), explored the relationships between phonemic awareness, reading fluency, and articulatory ability in three groups of French-speaking university students, each of which represented a particular level of competence in these three types of abilities. The hypotheses partly drew on Liberman’s [18] theory according to which articulatory gestures constitute the information underlying phoneme representation. As such, articulatory gestures are likely to influence reading abilities and reading-related competencies such as phonemic awareness. The research plan included two groups (N = 52) of dyslexic participants, one with dyslexia accompanied by motor impairment while the other had only dyslexia, and a third group (N = 59) of skilled readers. They were administered a reading task, a phonemic awareness task, and an articulatory task assessing the three major orofacial articulatory organs (lips, tip of the tongue, and dorsum of the tongue). 

The results showed that both dyslexic groups had significantly poorer reading abilities than the controls, as expected. In phonemic representation, however, some differences were found between the two dyslexic groups in favor of the less impaired subgroup, suggesting that dyslexia with articulatory impairment added further difficulty in phonemic representation. A very similar pattern also appeared in the results on articulatory control, where speech timing was worse in the subgroup of dyslexia with comorbidity. Further analyses showed that the articulatory rate explained phonemic awareness scores in both the skilled and dyslexic groups. To sum up, this ingenious research plan afforded the possibility to go beyond the already-known association between phonemic awareness and reading abilities. By exploring the impact of motor impairment in a specific subgroup of dyslexics, the authors showed that the motor deficits of these participants generalized to their speech-processing system, as revealed by their poor articulatory performance. As these same participants also had a worse performance in the phonemic awareness task, these outcomes would suggest that respiratory control and pneumo-phonic coordination affect phonemic representation, which in turn affects reading abilities. The overall result is not only relevant on theoretical grounds but also confirms the value of already implemented training for dyslexia remediation that reduces phonological disorder and improves spelling and reading skills. 

The other article on dyslexia, from two research institutes in Genoa (Italy) (Contribution 2), investigated the relationship between reading abilities and a general cognitive factor such as proportional reasoning in 7- to 9-year-old dyslexic children and a control group of skilled readers, matched by age, gender distribution, and general intelligence. The central idea is that one of the main requirements of reading is to segment written sequences into parts at different scales, such as words and syllables, and that this ability relies on a more profound cognitive ability to segment a continuum into discrete units and perceive the whole–parts relationships, which is precisely what is required in proportional reasoning. The child was presented with a drawing showing a column with a given proportion of water and juice, and was asked to choose from two alternative figures the one which represented the same proportions as the target beyond differences in absolute values. To verify the possible influence of continuous versus discrete forms of presentation of the stimuli, the authors devised conditions where both the target and the alternatives were drawn as segmented blocks (discrete/discrete: D-D), followed by others where they appeared as non-segmented blocks (continuous/continuous: C-C), and others where the discrete/continuous condition was mixed (either C/D or D/C). A series of ANOVAs showed a main effect of age, with younger children performing worse than the older ones, but also a main effect of the clinical condition, with dyslexic children performing significantly worse than the controls. Correlations, on their side, showed that the worse the performance at the DC condition on the proportional reasoning task, the worse the accuracy in reading words, non-words, and text. Based on these arguments, the authors confirm their hypothesis of a link between dyslexia, on the one hand, and a more general impairment in processing the metrical structure of stimuli, on the other, which might be transversal across different domains (arithmetic, music, language).

The articles centered on people with WS addressed two interrelated aspects of language competence, one having to do with the relationships between lexical knowledge and semantic comprehension and the other with a relevant pragmatic ability extensively studied by psychologists as “mentalizing ability” (that is, the capability to infer the other’s thoughts). Both articles are from scholars of Hunan University (China). In one of the studies, the authors (Contribution 3) investigated the ability of children with WS to categorize emotions presented in the verbal form. This research explores the typical difficulty of this population in processing basic emotions and their valences (positive, negative, or neutral), going a step forward with respect to previous studies, and as such this study represents a double challenge. On the one hand, children with WS are known to have a rich vocabulary and to be particularly fluent and hypersocial, which would suggest that they have no problems with word production and comprehension. On the other, the studies that indicated atypical emotional recognition in this population were mostly based on visual cues but failed to explore emotion comprehension at a deeper level when the task requires matching words on the basis of emotional meaning. The present study intended to fill this gap by means of an emotion-priming task. Three types of emotional stimuli, with positive, neutral, and negative emotional valence, were presented to 14 children with WS, matched to 28 typically developing children, half of which were matched on mental age (MA) and the other half on chronological age (CA), and of general intelligence. Each emotional valence was used as a prime and matched to a target in one of the same types of emotional valence. All participants were asked to judge whether the emotional valence of the prime and the target were congruent. The authors hypothesized that the priming effect would be weaker for the same emotional valence in people with WS. Participants with WS showed patterns of emotion priming fundamentally similar to those of the controls but with lower accuracy. For instance, under neutral and negative prime conditions, the participants with WS confused positive and neutral emotion targets as they perceived them as being similar. Combining these outcomes with those of previous studies that highlighted difficulties in integrating propositions [19] and causal inferences [20] in people with WS, the authors concluded that this clinical population has only a superficial understanding of emotional words and, generally speaking, a poor analysis of the meanings of words, and therefore poor meta-semantic abilities.

The other study on WS (Contribution 4) also addressed an important aspect of social cognition and interpersonal communication in this population, namely the capability to interpret the other’s mind, which goes under the name of Theory of Mind. This capability demands the interpretation and integration of multiple cues, both linguistic and visual. As both these cues might be misinterpreted by children with WS, the authors devised a computerized format of the standard “false belief” tasks assessing Theory of Mind. The hypothesis was that this format could help these children overcome their interpretation difficulties, and thereby improve their mentalizing abilities. Twenty-two children with WS (CA = 9.9; MA = 6.4) were administered the unexpected location-change task, and seventeen children with WS (CA = 10.3; mean MA = 6.7) were administered the unexpected content-change task under the form of animated cartoons. False belief tasks were passed by children with WS at an earlier age than generally reported (CA = 5.9 years vs. 9 years), suggesting that the computerized format devised by the authors enhanced their mentalizing ability to a certain extent. This improvement opens up new pathways in which to explore interpretative processes in children with WS as well as to devise training programs to enhance these same processes.

In all four studies described so far, we always encountered some impaired forms of awareness and inferential processes in children or young adults belonging to two exemplar categories of developmental disorders: phonemic awareness and inferential mathematical reasoning in dyslexics, and meta-semantic awareness and mentalizing of the other’s thoughts in children with WS. But we may ask how typically developing children construct those inferential processes and come to more satisfactory forms of awareness of language usages or, better said, metalinguistic awareness. This is precisely the focus of Melogno’s, Pinto’s, and Lauriola’s study (“Sapienza” University of Rome and “Unicusano” University of Rome-Italy) (Contribution 5), which addresses the qualitative changes that normally take place when coping with metalinguistic issues in a relevant transitional phase, that between kindergarten and primary school. The authors conducted research on 158 typically developing 5- to 7-year-old participants, Italian monolinguals, with average socio-economic status, with no cognitive nor neurological deficit, and standard school achievement. Six metalinguistic tasks especially targeted to this age range were administered together with the Colored Progressive Matrices, one lexical and one grammatical comprehension test. A previous study [21] had already revealed a significant increase in all the linguistic, metalinguistic, and nonverbal intelligence measures across ages, correlations between these scores, and strong homogeneity between the metalinguistic scores. In the current study, the authors conducted a fine-grained analysis of how the cognitive levels underlying the global score of each metalinguistic task evolved year after year. Each level expresses a strategy to face the type of metalinguistic conflict that a given task poses. The results highlighted a significant evolution from the poorest strategy, which fundamentally eludes the conflict and prevails at 5 years, to the more articulated strategies where the conflict is gradually acknowledged and recomposed, which gradually appeared by ages 6 and 7. One of the interesting points made by this contribution is that the developmental trajectory of metalinguistic abilities does not consist of a transition from totally absent to fully achieved metalinguistic awareness. What the authors underline, on the contrary, is the gradual transition from a type of language processing entirely based on concrete, extra-linguistic arguments to processing language forms and structures per se, irrespective of their content, which is precisely the transition toward a metalinguistic plane. In addition, previous studies showed [22] that this graduality in metalinguistic processing is also present in more advanced developmental phases. The description of these gradual steps, in turn, can help us to better grasp the nature of the linguistic difficulties encountered by children with NNDs, and on these grounds guide these children toward more abstract language processing. 

A relevant form of metalinguistic awareness is constituted by the ability to reflect on meanings, technically called meta-semantic ability, which can be modulated as a function of the difference between literal and nonliteral meanings. Figurative language, in its multiple forms (metaphor, metonymy, idioms, irony, etc.) entirely relies on nonliteral meanings, and children’s capability to go beyond literal interpretations is a developmental process that has been extensively studied over the past six decades [23,24,25,26,27,28,29]. In this Special Issue, the article by Fanari, Melogno, and Fadda (University of Cagliari, “Sapienza”—University of Rome-Italy) (Contribution 6) addresses one of these forms, namely sarcasm, and describes two studies on school-age children’s comprehension of sarcastic expressions. The first study aimed to verify and extend a previous study by Massaro et al. [30], which explored sarcasm comprehension in children in relation to the Theory of Mind, finding a correlation between these two variables. Fifty-five Italian typically developing fourth graders were administered irony tasks, Digit span forward and backward, the Colored Raven’s Matrices, and a false belief task. While there were no correlation between the irony, the cognitive, and the Theory of Mind measures, significant differences appeared among the irony task measures as a function of contextual variations. Children better understood sarcastic expressions in stories where the conversational partner had both a high level of authority and familiarity (the mother) rather than when it was an adult with a lower level of both authority and familiarity (the cashier of a food store). A further and more extended study was conducted on a wider sample (N = 180) with first, third, and fifth graders. Again, neither Theory of Mind nor cognitive factors proved to have an influence on sarcasm understanding, while the major factors were age and, once more, the same contextual factors were revealed by the first study. The confirmed dominance of this contextual dimension over cognitive and meta-representational factors highlights the pragmatic nature of sarcasm understanding, which, therefore, is not solely a semantic ability. Various studies have indicated this pragmatic aspect in other forms of figurative language comprehension as well (for a review, see [23]), both with typically and atypically developing children. Other studies have also reported the outcomes of training experiences to enhance this type of language comprehension [31,32,33,34,35].

## 3. Conclusions

Each of the studies reported in this editorial sheds light on aspects previously unexplored of the connections between language competencies and NDDs. This obliges us to consider the role of some components within overall cognitive or linguistic competencies in both typically and atypically developing children, which, in turn, has consequences on our redefinition of a whole range of NDD profiles. On these grounds, moreover, we can conceive training programs likely to empower those aspects of language that have been highlighted as weaknesses in some or all the NDDs described, the outcomes of which can further illuminate the specificities of these disorders.
